# Melanoma Marjolin ulcer in residual limb of patient of color with below-knee amputation

**DOI:** 10.1016/j.jdcr.2024.07.009

**Published:** 2024-07-27

**Authors:** Joshua Siar, Dimple Patel, Theresa Rohr-Kirchgraber

**Affiliations:** Medical College of Georgia, Augusta University University of Georgia Medical Partnership, Athens, Georgia

**Keywords:** amputation, below-knee amputation, Marjolin ulcer, melanoma, melanoma on black skin, pathology, scar tissue carcinoma, skin of color

## Introduction

Melanoma develops when melanocytes undergo malignant changes, leading to tumor formation.^1^ Melanoma is most often found in White individuals and risk factors for the development of melanoma include family history, pale complexion, low socioeconomic status, immunosuppression, and sun exposure.^1^

Skin cancers arising from chronic wounds are termed Marjolin ulcers. Over 95% of these ulcers manifest as squamous or basal cell carcinomas, whereas melanomas constitute only 2.5% of Marjolin ulcers.^2^ Here, we present the case of a 72-year-old man with a history of a below-knee amputation (BKA) who presented with suspected infection later diagnosed as an ulcerated malignant melanoma originating from the scar tissue of his residual limb.

## Case report

**Fig 1:**
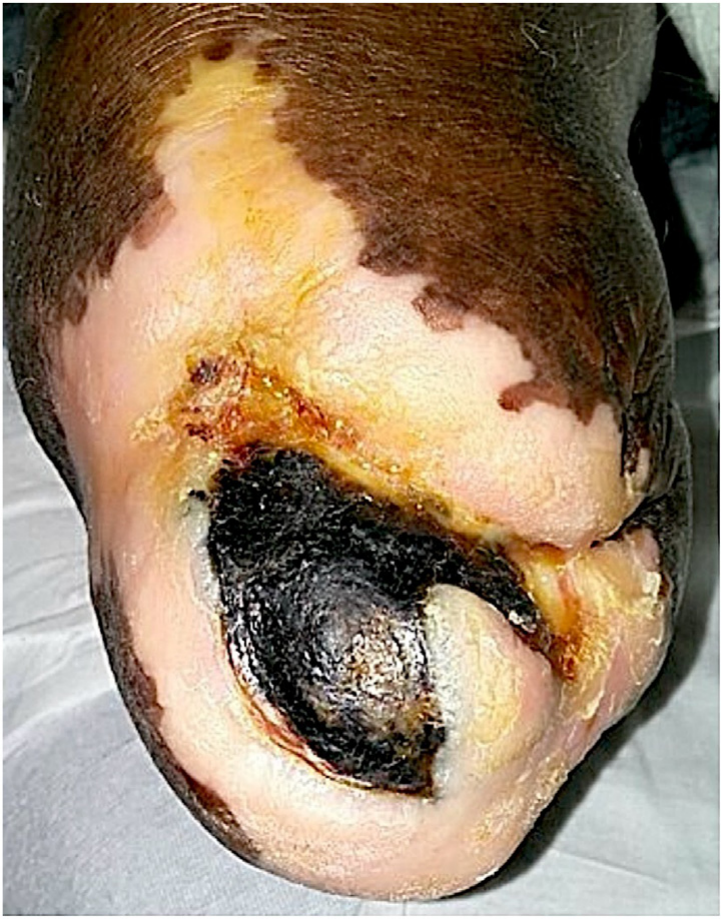
Residual limb prior to excisional biopsy demonstrating malignant melanoma.

A 72-year-old Black man, with a history of right BKA stemming from a motor vehicle accident at age 9 years, presented initially with concerns of a wound infection at the site of his residual limb. His medical history included hypertension and diabetes. Upon presentation, he exhibited symptoms of a malodorous infection in his right leg. Before this admission, the patient had not experienced any ulcer wounds in this area until approximately 3 months earlier. Notably, he had recently begun using a new prosthetic limb, alternating with the old one because of discomfort. About a week before presentation, a scab over the wound on his right limb had detached while showering, revealing black material and serous drainage beneath. This drainage persisted for 1 month, without any accompanying systemic symptoms. Upon admission, magnetic resonance imaging tibia and fibula indicated signs suggestive of cellulitis without abscess or clear evidence of osteomyelitis. Considering the concern for a wound infection, orthopedics recommended a revision of the BKA, with incision and drainage of the wound. Standard protocol dictated the submission of bone and tissue samples for pathology examination. The final pathologic report of the wide local excision unveiled an unexpected finding: an ulcerated malignant melanoma originating within the residual limb, measuring 6 mm in Breslow thickness and 3 cm in its greatest dimension, classified as Clark level V. All margins were negative for invasive melanoma. Regression was present, however, the depigmentation noted in Fig 1 represents a region of vitiligo. A sentinel lymph node biopsy was not accomplished by orthopedics at the time of excision but follow up positron emission tomography scan and core needle biopsy demonstrated 2 positive lymph nodes, 1 in the inguinal region, and 1 in the axilla. The patient was referred to oncology and is being treated with ipilimumab monotherapy.

## Discussion

The relationship between chronic wounds and malignancy can present in a multitude of ways. Chronic wounds may transform into malignancy, although conversely, primary malignancies may initially present as wounds.^3^ Distinguishing between primary malignant ulcers from malignant transformation of chronic wounds is often difficult. Considering the patient’s 63-year history of BKA with associated irritation to the residual limb from long-term prosthetic use, the carcinoma’s etiology was suspected to be secondary to many years of chronic inflammation with an ulcer of 3 months duration.

The pathogenesis behind skin cancer emerging from wounds remains multifaceted and is yet to be fully understood. At present, the prevailing hypothesis involves excess cell proliferation triggered by chronic inflammation within scar tissue.^4^ This inflammatory cascade dampens cell-mediated immunity and fosters angiogenesis, cellular proliferation, and the suppression of apoptosis, all cellular hallmarks of carcinogenesis.^5^ Moreover, residual limb sites are fragile cutaneous landscapes and when this susceptible tissue is placed into a prothesis, it must adapt to a humid environment and also withstanding both frictional and compressive forces for which it is not well adapted.^6^ These factors make residual limb tissue more prone to tissue breakdown causing an area of local immune dysregulation referred to as an immunocompromised district, predisposing scar tissue to malignant transformation.^6^^,^^7^ Basal and squamous cell carcinomas along with lymphangiosarcomas have all been reported to affect residual limbs, but there are few if any known reported cases of melanoma on residual limbs in the literature, thus making this case exceptionally uncommon.^6^

This case underscores the importance of thorough evaluations for wounds of uncertain origin, emphasizing the necessity of routine pathology examination of tissue as a crucial aspect of the diagnostic process when a definitive clinical diagnosis is lacking.^8^ Current guidelines recommend wound biopsy if a lesion demonstrates increased size, malodor, and pain; excessive granulation tissue, bleeding, or drainage; exophytic growth; or irregular base or margin, many criteria our patient’s wound met. It is noteworthy that previous case reports of carcinomas on residual limbs of patients with amputations exhibit metastatic potential.^8^ One case of verrucous hyperplasia on a patient’s residual limb developed into squamous cell carcinoma and metastasized to bone and lung.^9^ Our case is a critical reminder of the importance of prompt pathologic evaluation of tissue from amputation-site ulcers meeting criteria for wound biopsy. Without the applied proactive approach, the diagnosis of melanoma in this patient may well have been overlooked, thus putting the patient at increased risk of advanced disease.

Although melanoma is more common in individuals with white skin, those with skin of color often face poorer survival rates because of various factors. These include limited education about skin cancer in communities of color, lower screening rates, socioeconomic obstacles, a higher prevalence of aggressive melanoma subtypes, and inadequate representation in medical research and education.^10^ Although the prompt recognition of melanoma in this case is noteworthy, this report stands as a distinctive illustration of melanoma in a person of color with a BKA, serving as a poignant reminder of the diverse manifestations of melanoma across different skin tones. Ultimately, this case underscores the possible diagnosis of scar tissue melanoma in patients with a BKA, and also emphasizes the importance of pathology evaluation of all tissue in wounds of unknown origin.

## Conflicts of interest

None disclosed.
